# Long-Term CD4+ Cell Count in Response to Combination Antiretroviral Therapy

**DOI:** 10.1371/journal.pone.0093039

**Published:** 2014-04-02

**Authors:** Paula M. Luz, Beatriz Grinsztejn, Luciane Velasque, Antonio G. Pacheco, Valdilea G. Veloso, Richard D. Moore, Claudio J. Struchiner

**Affiliations:** 1 Instituto de Pesquisa Clínica Evandro Chagas, Fundação Oswaldo Cruz, Rio de Janeiro, Brasil; 2 Departamento de Matemática e Estatística, Universidade Federal do Estado do Rio de Janeiro, Rio de Janeiro, Brasil; 3 Department of Medicine, Johns Hopkins University, Baltimore, Maryland, United States of America; 4 Programa de Computação Científica, Fundação Oswaldo Cruz, Rio de Janeiro, Brasil; University of Cape Town, South Africa

## Abstract

**Objective:**

There is a continuous debate on how to adequately evaluate long-term CD4+ cell count in response to combination antiretroviral therapy (ART) among human immunodeficiency virus (HIV)-infected individuals. Our study evaluated the long-term CD4+ cell count response (up to ten years) after initiation of ART and described the differences in the CD4+ cell count response stratified by pretreatment CD4+ cell count, and other socio-demographic, behavioral, and clinical factors.

**Methods:**

The study population included patients starting ART in the clinical cohorts of Rio de Janeiro, Brazil, and Baltimore, United States. Inverse probability of censoring weighting was used to estimate mean annual CD4+ cell counts while adjusting for choice of initial ART regimen, ART discontinuation and losses-to-follow-up.

**Results:**

From 1997 to 2011, 3116 individuals started ART; preferred initial regimen was NNRTI-based (63%). The median follow-up time was 5 years, 10% of the individuals had nine or more years of follow-up. Observed CD4+ cell counts increased throughout the ten years of follow-up. Weighted results, in contrast, increased up to year four and plateaued thereafter with 50% of the population reaching CD4+ cell counts of 449/μL or more. Out of all stratification variables considered, only individuals with pre-treatment CD4+ cell counts ≥350/μL showed increasing CD4+ cell counts over time with 76% surpassing the CD4+ cell count >500/μL threshold at year ten.

**Conclusion:**

The present study corroborates the growing body of knowledge advocating early start of ART by showing that only patients who start ART early fully recover to normal CD4+ cell counts.

## Introduction

Combination antiretroviral treatment (ART) can suppress human immunodeficiency virus (HIV) RNA and allow for immune restoration. Poor immune restoration (<500 CD4+ cells/μL) leads to increased morbidity and mortality from AIDS-related illnesses and also from non-AIDS related conditions [1]–[4]. A recent study reviewed results from cohort studies and showed that improvements in CD4+ cell counts persist up to 7–10 years after initiation of ART in high-income countries [5]. Results from low-middle-income countries could potentially be the same as those achieved in high-income counties though limitations regarding selection of patients as well as analyses were present [5].

Most studies of long-term CD4+ cell count response report results for only a fraction of the study population. Some studies restrict inclusion criteria for those with suppressed HIV RNA (for example, to <50 copies/ml) and are thus estimating optimal long-term CD4+ cell count response [6] while other studies do not adequately adjust for individuals who might have interrupted ART or have been lost to follow-up. The characteristics of patients who do not suppress HIV RNA, interrupt treatment and/or drop out are likely different from those who remain suppressed, under treatment and/or in care and these differences can lead to selection biases. A recent study accounted for both factors in the analysis through the use of inverse probability of censoring weighting and found that CD4+ cell count continued to show only modest increase between years three and seven in a cohort from the United States [7].

Moreover, further biases may arise if the tendency to prescribe an initial ART regimen to a particular type of patient is not accounted for. In many settings where ART is widely available, the initial ART regimen composed of three antiretroviral drugs can be either based on a non-nucleoside reverse transcriptase inhibitor (NNRTI) or a protease inhibitor (PI) drug class. The actual choice of a specific regimen for a particular patient is guided by many factors including patient's characteristics (socio-demographic and clinical, including co-morbidities), physician preference, knowledge and experience, pill burden, and drug availability and cost. That is, in cohort studies, an initial ART regimen is not assigned randomly to patients (as in clinical trials) and, thus, it may well be that a particular sub-group of patients are more likely to receive a particular type of initial ART regimen. If, for example, patients with severe immunodeficiency are initiated on a PI-based regimen more frequently, an analysis that does not adjust for type of initial ART regimen will incorrectly conclude that PI-based regimens lead to poorer CD4+ cell count response.

Our study evaluated the long-term CD4+ cell count response (up to ten years) after initiation of ART and described the differences in the CD4+ cell count response according to pretreatment CD4+ cell count, and other socio-demographic, behavioral, and clinical factors. We used inverse probability of censoring weighting (IPCW) to estimate annual CD4+ cell counts while adjusting for choice of initial ART regimen, ART discontinuation and lost to follow-up. Our evaluation was done in the context of two clinical cohorts situated in Rio de Janeiro, Brazil, and Baltimore, United States, where ART has been provided to HIV-infected individuals since 1996.

## Methods

### Ethics statement

This study was approved by the ethics committee of the Evandro Chagas Clinical Research Institute of the Oswaldo Cruz Foundation and the Johns Hopkins University School of Medicine Institutional Review Board, and it was conducted according to the principles expressed in the Declaration of Helsinki. Participants provided written informed consent.

### Description of the clinical cohorts

The Johns Hopkins AIDS Service provides care for a large proportion of HIV-infected patients in Baltimore. An observational, longitudinal, clinical database has been maintained on patients receiving primary HIV care since 1990. In this longitudinal database, data are updated regularly using outpatient and inpatient clinical documentation (from the Johns Hopkins AIDS Service and elsewhere), laboratory testing results, and pharmacy records. Prescription of antiretroviral therapy (drug, dates of use, and dose) is documented by the medical provider and support staff in the clinical records. Trained abstractors record all this information onto standardized forms for processing. Details of the methodology have been previously described [8].

The Evandro Chagas Clinical Research Institute (IPEC) AIDS Service has provided care to HIV-infected patients in Rio de Janeiro since 1986. An observational, longitudinal, clinical database has been maintained on patients receiving primary HIV care in the clinic since 1998. The data collection process was patterned after the process established at the Johns Hopkins AIDS Service. Longitudinal data are updated regularly using outpatient and inpatient clinical documentation, laboratory testing results, and pharmacy records. Prescription of antiretroviral therapy (drug, dates of use, and dose) is documented by the medical provider and support staff in the clinical records. Trained abstractors record all this information onto standardized forms for processing. Further details and results can be found in published studies [9].

### Inclusion criteria and outcome definition

We analyzed data on ART naïve HIV-infected adults (> = 18 years of age at enrollment) who enrolled in the Johns Hopkins and the IPEC clinical cohorts on/after January 01 1997. Only patients who started ART during the period from January 01 1997 through July 31 2011 were included in the present analysis and start of follow-up was defined as date of start of ART. Follow-up extended to December 31 2011 and thus patients starting ART up to December 31 2001 had a potential follow-up of ten years under treatment while patients starting ART after this date contributed up to their possibilities. For those who died, end of follow-up was defined as the year of death. For those not known to have died, end of follow-up was given by the last year when a CD4+ cell count was available. If this year was the patient's tenth year of follow-up, then administrative censoring was applied and follow-up was assumed complete. In contrast, if this year was not the patient's tenth year of follow-up, then the patient was assumed lost to follow-up for the subsequent years up to year 10 (i.e. informative censoring). Throughout a patient's follow-up time, years for which CD4+ cell counts were not available were treated as missing data. The outcome of interest was a patient's annual CD4+ cell count, defined as the mean of the CD4+ cell counts done during the actual year since start of ART.

### Statistical analyses

We describe the observed (unweighted) and corrected (IPCW-weighted) annual CD4+ cell counts, as represented by the median and interquartile range values, for patients since start of ART. Corrected CD4+ cell counts are defined as those adjusted for informative censoring due to choice of initial ART regimen, treatment discontinuation, and lost to follow-up, using IPCW [10]. For a complete description of the method as it relates to providing corrected long-term CD4+ cell counts please refer to [7]. Briefly, by allocating weights to patients on follow-up based on their characteristics, IPCW allows for a description of the entire study population (instead of a subgroup that, for diverse reasons, was selected over time). Thus, though outcomes might not be available for a fraction of the patients, these are represented by increasing the weight given to similar patients for whom the outcome is available. Weights are derived from regression models, described below, used to identify patients that are similar to each other based on the available covariates.

Weights were obtained using logistic regression models to determine which socio-demographic and clinical factors explained the probability of discontinuing ART (defined as ART discontinuation for more than 60 days), the probability of being lost to follow-up (as defined above), and the probability of being prescribed PI-based regimen as opposed to an NNRTI-based regimen. Unadjusted analyses were performed and factors found to be associated with the outcome at the threshold significance of 0.2 were included in the initial multivariate model. Final models were determined based on threshold significance of 0.05, clinical relevance and lowest Akaike information criterion. The interaction of cohort with covariates present in the final model were tested and maintained if statistically significant. Factors included in the logistic regression models were those that we considered might be confounding variables, that is, predict either treatment discontinuation, lost to follow-up, initial ART regimen and also long-term CD4+ cell count. These included site (Rio de Janeiro or Baltimore), age, gender, race/ethnicity, injection drug use (IDU), pretreatment CD4+ cell count and viral load, nadir CD4+ cell count, initial ART regimen, AIDS defining illness (ADI) at the start of ART, and hepatitis B and C co-infections. R statistical software (www.r-project.org version 2.15.2) and packages IPW and quantreg were used for all analyses.

## Results

Overall, 3116 patients were included in the present analysis, 1822 (58.5%) from IPEC and 1294 (41.5%) from the Johns Hopkins clinical cohorts (Table 1). The median age at the start of ART was 39 years, 16.4% were aged 50 years or more. Males represented two-thirds of the population, 63.2% of the participants were non-white. IDU was reported by 13% of the participants. Pre-treatment CD4+ cell count was low (median of 194 cells, interquartile range [IQR] 65–299), nadir CD4+ cell count was somewhat lower (median [IQR]: 179 [53–279] cells/μL), and 32.3% of the individuals had pretreatment HIV RNA >100,000 copies/mL. The preferred initial regimen was NNRTI-based (62.9%), and the prevalence of ADI and hepatitis C co-infection were, respectively, 33.2% and 15.3% (Table 1).

**Table 1:** Demographic, behavioral and clinical characteristics at the start of ART for patients followed at the Evandro Chagas Clinical Research Institute (IPEC) and the Johns Hopkins AIDS Services.

	IPEC	Johns Hopkins	Total
Patients	1822	1294	3116
**Age** a			
Median (IQR)	36 (30,44)	42 (36,48)	39 (32,46)
<30 years	421 (23.1)	130 (10)	551 (17.7)
30–39 years	684 (37.5)	377 (29.1)	1061 (34.1)
40–49 years	490 (26.9)	504 (38.9)	994 (31.9)
>50 years	227 (12.5)	283 (21.9)	510 (16.4)
**Gender**			
Female	573 (31.4)	469 (36.2)	1042 (33.4)
Male	1249 (68.6)	825 (63.8)	2074 (66.6)
Race/ethnicity			
White	905 (49.7)	238 (18.4)	1143 (36.7)
Non-white	917 (50.3)	1056 (81.6)	1973 (63.3)
**HIV risk exposure category** b			
Not IDU	1807 (99.2)	904 (69.9)	2711 (87)
IDU	15 (0.8)	390 (30.1)	405 (13)
**Pre-treatment CD4 T-cell count** c			
Median (IQR)	199 (74,292.8)	184 (51.2,314)	194 (65,299)
<100	479 (26.3)	340 (26.3)	819 (26.3)
100–199	306 (16.8)	186 (14.4)	492 (15.8)
200–349	556 (30.5)	273 (21.1)	829 (26.6)
> = 350	221 (12.1)	199 (15.4)	420 (13.5)
Missing	260 (14.3)	296 (22.9)	556 (17.8)
**Nadir CD4 T-cell count**			
Median (IQR)	190 (71.5,278)	158 (32.8,280)	179 (53,279)
<50	311 (17.1)	346 (26.7)	657 (21.1)
50–199	528 (29.0)	327 (25.3)	855 (27.4)
200–349	606 (33.3)	312 (24.1)	918 (29.5)
> = 350	170 (9.3)	159 (12.3)	329 (10.6)
Missing	207 (11.4)	150 (11.6)	357 (11.5)
**Pre-treatment HIV viral load** c			
≤400	30 (1.6)	120 (9.3)	150 (4.8)
401–3000	59 (3.2)	55 (4.3)	114 (3.7)
3001–10000	117 (6.4)	80 (6.2)	197 (6.3)
10001–100000	527 (28.9)	395 (30.5)	922 (29.6)
>100000	625 (34.3)	382 (29.5)	1007 (32.3)
Missing	464 (25.5)	262 (20.2)	726 (23.3)
**Initial ART regimen** d			
NNRTI	1280 (70.3)	679 (52.5)	1959 (62.9)
PI	505 (27.7)	587 (45.4)	1092 (35)
**ADI at start of ART** e			
No	1061 (58.2)	1019 (78.7)	2080 (66.8)
Yes	761 (41.8)	275 (21.3)	1036 (33.2)
**Hepatitis B co-infection** f			
No	1750 (96)	1231 (95.1)	2981 (95.7)
Yes	72 (4.0)	63 (4.9)	135 (4.3)
**Hepatitis C co-infection** f			
No	1746 (95.8)	892 (68.9)	2638 (84.7)
Yes	76 (4.2)	402 (31.1)	478 (15.3)

HIV: human immunodeficiency virus, ART: antiretroviral therapy, ADI: AIDS defining illness.

a Age at the start of ART.

b Reported mode of HIV risk exposure was categorized injection drug users (IDU) and not IDU

c Pre-treatment CD4+ cell count and HIV RNA were defined as the value closest to the date of start of ART up to 6 months prior.

d Initial ART regimen was classified as NNRTI-based or PI-based. Integrase inhibitor-based regimens were too few (N = 75) to draw consistent conclusions and were thus excluded.

e Concurrent AIDS defining illness (ADI) was defined as the presence of any CDC 1993 condition at six months prior to up to one month after start of ART

f Hepatitis B/C co-infection was defined as having chronic infection at the start of ART.

The mean follow-up time was 4 years (median 5 years, IQR 2–6), 241 (7.7%) of the individuals had ten years of follow-up. Discontinuation of ART was observed for 832 patients (832/3116, 26.7%). Site (Johns Hopkins), non-white race, higher pre-treatment viral load, PI-based regimen and Hepatitis C co-infection were found to significantly increase the odds of discontinuing ART (Table 2). In contrast, older age at the start of ART, nadir CD4+ cell count > = 350 cells/μL) significantly decreased the odds of discontinuing ART. Loss to follow-up was observed for 817 (26.2%) patients. Covariates found to significantly increase the odds of being lost to follow-up included site (Johns Hopkins), higher pre-treatment viral load, and hepatitis C co-infection, while nadir CD4+ cell count >50 cells/μL decreased the odds of being lost to follow-up. Female sex, higher pretreatment CD4+ cell count and pretreatment AIDS defining illness decreased the odds of having a PI-based regimen prescribed.

**Table 2:** Column 2: Demographic, behavioral and clinical characteristics of the study population (number [percentages] are given unless otherwise stated); Columns 3 to 8: Results of the logistic regression models for the three outcomes: ART discontinuation, Loss to Follow-up and Initial ART Regimen.

		ART Discontinuation		Loss to Follow-up		Initial ART Regimen	
		Unadjusted	Adjusted	Unadjusted	Adjusted	Unadjusted	Adjusted^h^
	N (%)	OR (95%CI)	OR (95%CI)	OR (95%CI)	OR (95%CI)	OR (95%CI)	OR (95%CI)
**Total**	3116						
**Cohort**							
IPEC	1822 (58.5)	Ref.	Ref.	Ref.	Ref.	Ref.	Ref.
Johns Hopkins	1294 (41.5)	5.99 (5.02, 7.15)	5.75 (4.68, 7.07)	5.10 (4.28, 6.08)	4.76 (3.94, 5.75)	2.19 (1.88, 2.55)	1.63 (1.35, 1.96)
**Age** a							
Median (IQR)	39 (32,46)						
<30	551 (17.7)	Ref.	Ref.	Ref.		Ref.	
30–39	1061 (34.1)	1.34 (1.06, 1.71)	0.99 (0.76, 1.29)	1.54 (1.20, 1.98)		1.06 (0.85, 1.32)	
40–49	994 (31.9)	1.43 (1.12, 1.83)	0.73 (0.55, 0.97)	1.54 (1.20, 1.99)		1.20 (0.96, 1.49)	
50+	510 (16.4)	0.95 (0.71, 1.27)	0.43 (0.31, 0.60)	1.79 (1.34, 2.38)		1.22 (0.95, 1.58)	
**Gender**							
Female	1042 (33.4)	0.81 (0.68, 0.96)		1.05 (0.88, 1.24)		0.54 (0.46, 0.63)	0.55 (0.47, 0.64)
Male	2074 (66.6)	Ref.		Ref.		Ref.	Ref.
**Race/ethnicity**							
White	1143 (36.8)	Ref.	Ref.	Ref.		Ref.	
Non-white	1966 (63.2)	2.21 (1.84, 2.64)	1.26 (1.03, 1.55)	1.72 (1.44, 2.05)		1.25 (1.07, 1.46)	
**HIV risk exposure category** b							
Not IDU	2711 (87)	Ref.		Ref.		Ref.	
IDU	405 (13)	3.27 (2.64, 4.06)		3.46 (2.79, 4.30)		1.80 (1.46, 2.23)	
**Pre-treatment CD4 T-cell count** c							
Median (IQR)	194 (65,299)						
<100	819 (26.3)	Ref.		Ref.		Ref.	Ref.
100–199	492 (15.8)	0.91 (0.70, 1.17)		0.80 (0.62, 1.03)		0.80 (0.63, 1.01)	0.72 (0.56, 0.92)
200–349	829 (26.6)	0.73 (0.58, 0.91)		0.61 (0.49, 0.77)		0.72 (0.58, 0.88)	0.62 (0.49, 0.77)
350+	420 (13.5)	0.83 (0.63, 1.09)		0.77 (0.59, 1.01)		0.95 (0.75, 1.22)	0.77 (0.59, 1.00)
Missing	556 (17.8)	1.21 (0.95, 1.53)		1.25 (0.99, 1.57)		0.96 (0.77, 1.21)	0.84 (0.66, 1.06)
**Nadir CD4 T-cell count**							
Median (IQR)	179 (53,279)						
<50	657 (21.1)	Ref.	Ref.	Ref.	Ref.	Ref.	
50–199	855 (27.4)	0.67 (0.53, 0.83)	0.93 (0.72, 1.19)	0.60 (0.48, 0.75)	0.74 (0.58, 0.94)	0.81 (0.66, 1.01)	
200–349	918 (29.5)	0.55 (0.44, 0.69)	0.83 (0.65, 1.08)	0.49 (0.39, 0.62)	0.71 (0.55, 0.91)	0.73 (0.60, 0.91)	
350+	329 (10.6)	0.57 (0.42, 0.77)	0.67 (0.48, 0.94)	0.62 (0.46, 0.84)	0.71 (0.51, 0.99)	0.98 (0.74, 1.29)	
Missing	357 (11.5)	0.92 (0.69, 1.21)	1.16 (0.84, 1.59)	0.74 (0.56, 0.98)	0.80 (0.58, 1.09)	0.79 (0.60, 1.04)	
**Pre-treatment HIV viral load** c							
< = 100000	1383 (44.4)	Ref.	Ref.	Ref.	Ref.	Ref.	
>100000	1007 (32.3)	1.10 (0.92, 1.33)	1.26 (1.01, 1.56)	1.16 (0.96, 1.40)	1.32 (1.07, 1.63)	1.00 (0.84, 1.18)	
Missing	726 (23.3)	1.19 (0.97, 1.46)	1.40 (1.10, 1.77)	1.35 (1.10, 1.66)	1.68 (1.33, 2.11)	0.87 (0.72, 1.06)	
**Initial ART regimen** d							
NNRTI	1959 (62.9)	Ref.	Ref.	Ref.		-	
PI	1092 (35)	1.75 (1.48, 2.06)	1.32 (1.11, 1.59)	1.28 (1.09, 1.51)		-	
**ADI at start of ART** e							
No	2080 (66.8)	Ref.		Ref.		Ref.	Ref.
Yes	1036 (33.2)	0.76 (0.64, 0.91)		0.96 (0.81, 1.14)		0.69 (0.59, 0.81)	0.53 (0.42, 0.67)
**Hepatitis B co-infection** f							
No	2981 (95.7)	Ref.		Ref.		Ref.	
Yes	135 (4.3)	1.06 (0.72, 1.56)		1.57 (1.09, 2.26)		0.66 (0.45, 0.98)	
**Hepatitis C co-infection** f							
No	2638 (84.7)	Ref.	Ref.	Ref.	Ref.	Ref.	
Yes	478 (15.3)	2.69 (2.19, 3.29)	1.44 (1.13, 1.82)	2.84 (2.31, 3.48)	1.45 (1.15, 1.81)	1.54 (1.26, 1.88)	

HIV: human immunodeficiency virus, ART: antiretroviral therapy, ADI: AIDS defining illness.

a Age at the start of ART.

b Reported mode of HIV risk exposure was categorized injection drug users (IDU) and not IDU.

c Pre-treatment CD4+ cell count and HIV RNA were defined as the value closest to the date of start of ART up to 6 months prior.

d Initial ART regimen was classified as NNRTI-based or PI-based. Integrase inhibitor-based regimens were too few (N = 75) to draw consistent conclusions and were thus excluded.

e ADI at the start of ART was defined as the presence of any CDC 1993 condition at six months prior to up to one month after start of ART.

f Hepatitis B/C co-infection was defined as having chronic infection at the start of ART.

h Significant cohort and ADI interaction term [aOR 2.02 (1.42, 2.86)].

**Figure 1:**
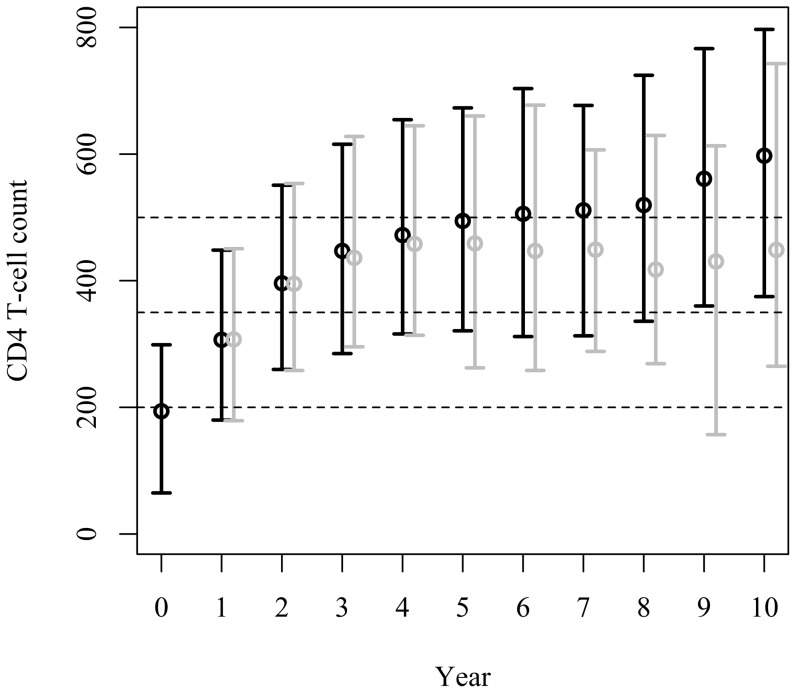
Observed (unweighted, black) and weighted (IPCW-weighted, gray) annual CD4+ cell counts (median and interquartile range) since start of ART. Dashed lines represent important CD4+ cell count thresholds of 200, 350, and 500 cells/μL.

Observed (unweighted) CD4+ cell counts display an increasing trend throughout the ten years of follow-up with values for the median and IQR reaching 307 (180–448), 472 (316–654), 511 (313–677), and 597 (375–797) cells/μL in years 1, 4, 7, and 10, respectively (Table 3 and Figure 1). The weighted results, on the other hand, show an increasing trend up to year 4 and a departure from this pattern thereafter (307 [179–451], 458 [314–645], 449 [288–797], and 449 [265–743] cells/μL at years 1, 4, 7, and 10, respectively, Table 3). Though observed data suggested that 50% of the population reached CD4+ counts of at least 597 cells/μL, the weighted results point in a different direction with 50% of the population reaching CD4+ counts of at 449 cells/μL or more and only 42.9% surpassing the >500 cells/μL threshold at year 10 (Table 3).

**Table 3:** Unstratified and stratified observed and weighted median (interquartile range) CD4+ cell counts for years 1, 4, 7 and 10 after start of antiretroviral therapy and the percentage of patients with weighted CD4+ cell count >500/μL at year 10.

	Year 1		Year 4		Year 7		Year 10		
	N = 3116		N = 1507		N = 576		N = 241		
	Observed	Weighted	Observed	Weighted	Observed	Weighted	Observed	Weighted	% CD4+>500/μL
**Unstratified**	307(180–448)	307(179–451)	472(316–654)	458(314–645)	511(313–677)	449(288–607)	597(375–797)	449(265–743)	42.9
**Cohort**									
IPEC	328(206–463)	315(186–459)	518(366–690)	446(289–646)	562(396–752)	420(282–588)	663(449–871)	434(265–764)	41.6
JH	264(141–414)	298(172–441)	414(230–594)	474(358–645)	456(209–633)	456(352–636)	486(293–705)	471(164–714)	45.4
**Age** a									
<50	309(182–450)	318(184–461)	483(325–663)	461(316–645)	515(313–692)	449(288–603)	602(387–824)	439(265–752)	41.2
50+	292(173–445)	290(166–408)	416(281–623)	452(301–654)	486(327–620)	496(270–620)	554(375–693)	627(318–694)	61.0
**Race/ethnicity**									
White	336(214–480)	328(197–477)	508(363–685)	494(352–672)	564(432–745)	477(352–663)	663(464–838)	554(275–783)	51.1
Non–white	283(163–421)	290(167–441)	440(282–632)	423(292–641)	463(238–655)	439(188–557)	511(313–752)	413(100–602)	33.9
**Gender**									
Women	313(194–460)	290(174–456)	500(330–696)	429(349–663)	532(310–742)	456(297–635)	628(404–797)	413(275–733)	42.6
Men	304(175–442)	314(182–449)	457(314–635)	466(311–644)	500(313–656)	416(288–593)	597(363–797)	452(230–777)	43.0
**HIV risk exposure category** b									
Not IDU	316(191–457)	316(186–456)	490(341–672)	481(314–672)	523(337–705)	446(288–635)	608(404–812)	466(275–774)	47.2
IDU	223(118–374)	242(133–401)	352(162–542)	366(315–567)	409(149–595)	456(353–456)	436(173–780)	173(80–471)	15.8
**Pre-treatment CD4 T-cell count** c									
<100	154(95–232)	158(102–235)	378(219–527)	366(218–496)	455(221–603)	456(307–503)	507(282–712)	466(127–648)	42.6
100–199	262(199–352)	266(200–334)	430(289–591)	395(282–590)	451(264–656)	288(126–496)	646(460–831)	413(80–481)	23.9
200–349	397(315–477)	392(307–477)	580(420–752)	541(396–718)	561(368–688)	448(335–660)	624(412–828)	539(387–856)	52.2
350+	546(457–676)	535(441–697)	644(477–852)	642(490–810)	709(532–890)	709(370–957)	818(586–1052)	**894(572**–**1079)**	**76.0**
Missing	249(144–398)	247(160–383)	440(292–580)	508(356–700)	478(313–617)	477(405–624)	521(347–746)	439(265–783)	43.9
**Nadir CD4 T-cell count**									
<50	137(74–218)	147(80–221)	362(174–518)	366(223–476)	443(200–596)	456(188–507)	504(293–728)	466(127–629)	43.3
50–199	262(187–352)	266(186–348)	434(291–597)	399(266–598)	496(269–670)	370(198–537)	628(374–797)	413(217–639)	31.0
200–349	409(332–501)	408(313–501)	594(431–759)	585(412–700)	591(405–720)	452(353–674)	657(434–859)	568(387–873)	52.2
350+	595(487–712)	630(508–752)	707(528–894)	680(543–820)	715(501–972)	654(545–998)	742(498–1069)	**732(428**–**1052)**	**67.5**
Missing	237(143–370)	217(141–339)	433(294–564)	495(325–700)	478(324–593)	449(324–581)	532(390–741)	439(265–783)	42.8
**Pre-treatment HIV RNA** c									
< = 100000	365(221–492)	360(213–485)	486(330–685)	440(315–644)	532(306–705)	456(282–636)	630(387–884)	507(217–873)	50.4
>100000	263(154–404)	281(144–424)	472(305–651)	481(289–641)	503(313–694)	370(277–593)	579(310–752)	413(230–647)	31.4
Missing	267(161–396)	267(168–406)	451(313–618)	496(322–700)	499(329–651)	452(352–592)	594(411–774)	508(317–783)	50.0
**Initial ART regimen** d									
PI-based	284(162–450)	300(179–460)	444(286–640)	446(319–652)	481(302–644)	379(283–585)	568(355–758)	441(265–738)	40.4
NNRTI-based	314(194–447)	326(186–449)	490(332–665)	466(314–645)	541(324–717)	456(288–624)	637(406–838)	471(208–752)	44.9
**ADI at the start of ART** e									
No	349(223–486)	352(238–492)	492(326–680)	510(350–685)	532(324–688)	449(282–654)	583(363–818)	449(230–743)	41.2
Yes	218(133–344)	204(136–337)	444(306–591)	376(240–565)	487(306–660)	448(294–515)	625(400–788)	466(411–738)	47.4
**Hepatitis C co**–**infection** f									
No	316(188–456)	310(181–448)	495(345–672)	475(314–663)	532(349–706)	449(288–644)	625(421–827)	476(275–783)	48.8
Yes	238(136–395)	298(167–498)	358(193–538)	366(320–589)	385(162–571)	456(198–456)	429(173–732)	317(80–471)	16.8

HIV: human immunodeficiency virus, ART: antiretroviral therapy, ADI: AIDS defining illness.

a Age at the start of ART.

b Reported mode of HIV risk exposure was categorized injection drug users (IDU) and not IDU.

c Pre-treatment CD4+ cell count and HIV RNA were defined as the value closest to the date of start of ART up to 6 months prior.

d Initial ART regimen was classified as NNRTI-based or PI-based. Integrase inhibitor-based regimens were too few (N = 75) to draw consistent conclusions and were thus excluded.

e ADI at the start of ART was defined as the presence of any CDC 1993 condition at six months prior to up to one month after start of ART.

f Hepatitis B/C co-infection was defined as having chronic infection at the start of ART.

**Figure 2:**
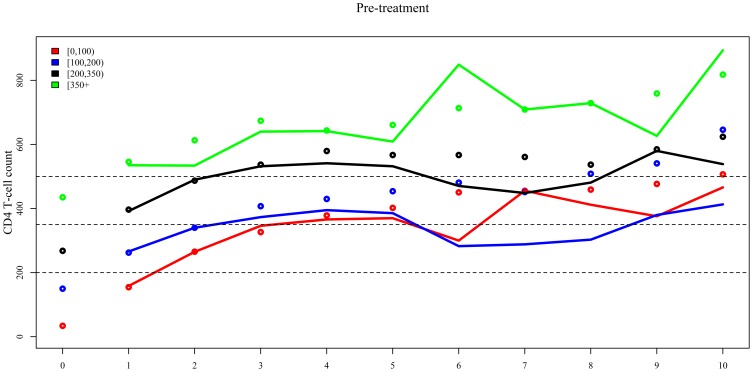
Observed (unweighted, dots) and weighted (IPCW-weighted, lines) annual median CD4+ cell counts since start of ART stratified by pre-treatment CD4+ cell count and nadir CD4+ cell count. Dashed lines represent important CD4+ cell count thresholds of 200, 350, and 500 cells/μL.

Up to year 4, observed and weighted median CD4+ cell counts show similar patterns for the three lowest CD4+ strata (observed and weighted medians [IQR] for year 4 by pre-treatment CD4+ stratum: <100 cells/μL 378 [219–527] vs. 366 [218–496]; 100–199 cells/μL 430 [289–591] vs. 395 [282–590]; and 200–349 cells/μL 580 [420–752] vs. 541 [396–718], Table 3 and Figure 2). Thereafter, weighted median CD4+ cell counts were consistently lower than the observed values. In contrast, for those with pre-treatment or nadir CD4+ cell counts ≥350 cells/μL, observed and weighted median CD4+ cell counts overlap over the study period. For the subgroup of individuals with pre-treatment CD4+ cell counts ≥350 cells/μL CD4+ cell counts continued to increase throughout the years, with 50% of the population reaching counts of at least 894 [IQR 572-1079] cells/μL at year 10 and 76% surpassing the >500 cells/μL threshold at year 10 (Table 3).

**Figure 3:**
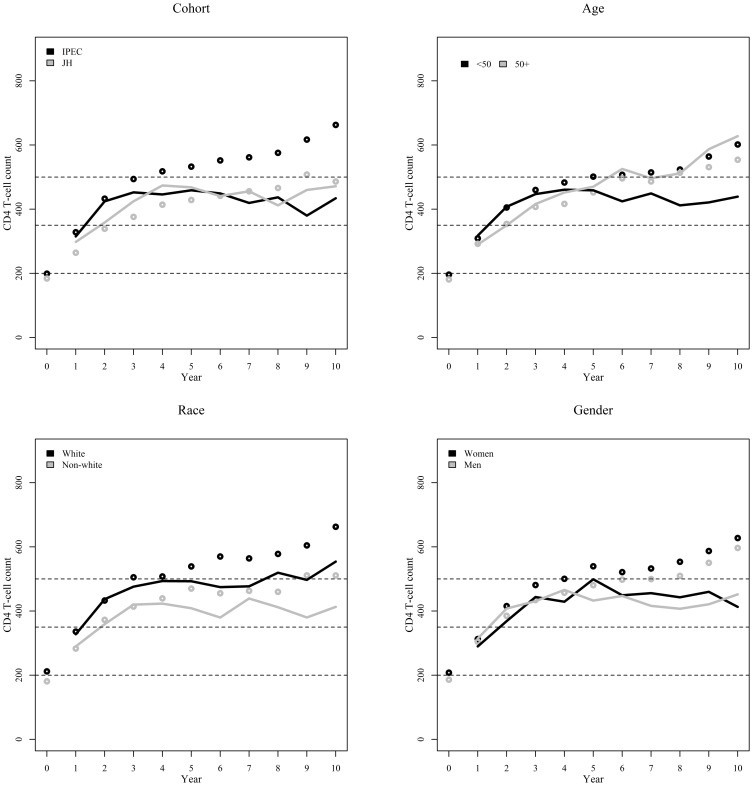
Observed (unweighted, dots) and weighted (IPCW-weighted, lines) annual median CD4+ cell counts since start of ART stratified by cohort, age at start of ART, gender and race. Dashed lines represent important CD4+ cell count thresholds of 200, 350, and 500 cells/μL.

Overall, though observed median CD4+ cell counts suggested increasing trends in the long-term immune response weighted results were more conservative except for the subgroup of individuals with 50 years or more (Table 3 and Figure 3). For individuals aged 50+ years, CD4+ cell counts increased over the years, with 61% of the individuals surpassing the >500 cells/μL threshold at year 10. Gender stratified results show, for both genders, that CD4+ cell counts increased up to year 4 and stable curves thereafter (Table 3 and Figure 3). For race, the weighting process lowered the median CD4+ cell count curves for whites and non-whites, without modifying the overall trend of improved median CD4+ cell counts for whites over time (Table 3 and Figure 3). Cohort stratified results show that the weighting process removes the apparent discrepancy in the observed median CD4+ cell counts, with both cohorts showing overlapping plateaus for the weighted results after year 4 (Table 3 and Figure 3).

**Figure 4:**
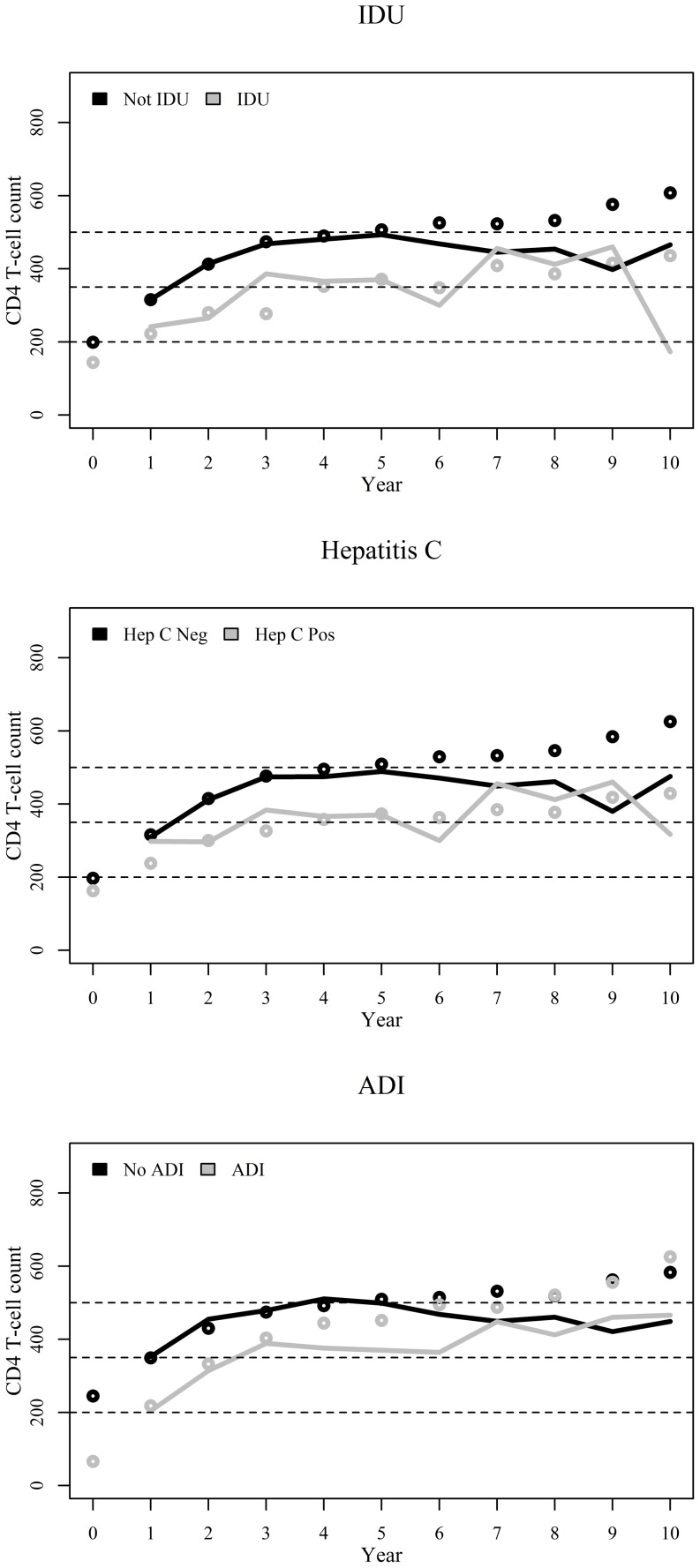
Observed (unweighted, dots) and weighted (IPCW-weighted, lines) annual median CD4+ cell counts since start of ART stratified by injection drug use (IDU), hepatitis C co-infection, and AIDS defining illness at the start of ART. Dashed lines represent important CD4+ cell count thresholds of 200, 350, and 500 cells/μL.

IDU stratified results were very similar to those for hepatitis C co-infection for which, up to year six, higher median CD4+ cells counts were shown for those not reporting injection drug use and not co-infected with hepatitis C (Table 3 and Figure 4). Weighted results show that only 16% of the individuals reporting IDU or with hepatitis C co-infected surpassed the >500 cells/μL threshold at year 10 (Table 3). Results stratified by concurrent ADI show improved median CD4+ cell counts up to year six for individuals without ADI though curves for both groups intermingle thereafter (Table 3 and Figure 4).

**Figure 5:**
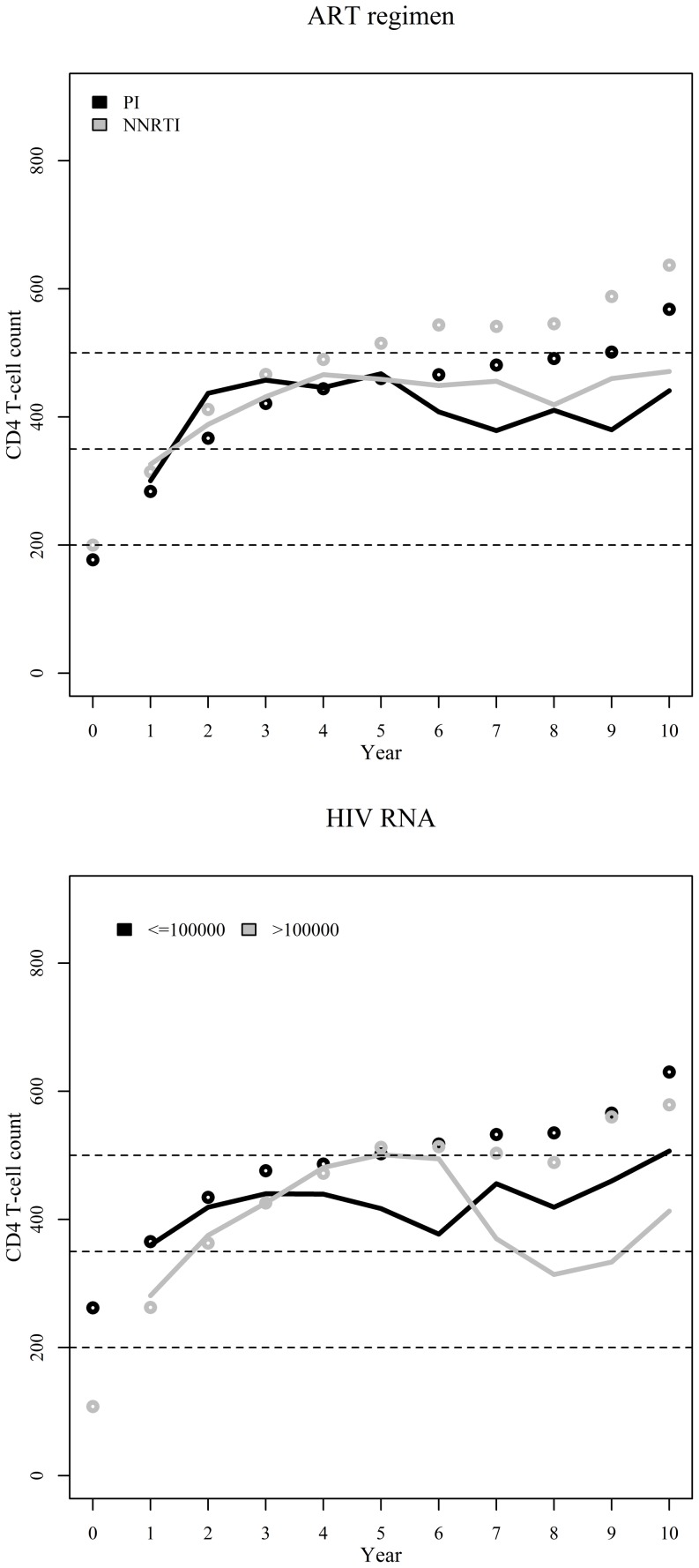
Observed (unweighted, dots) and weighted (IPCW-weighted, lines) annual median CD4+ cell counts since start of ART stratified by initial ART regimen and pre-treatment HIV RNA viral load. Dashed lines represent important CD4+ cell count thresholds of 200, 350, and 500 cells/μL.

Observed median CD4+ cell counts suggested a slight superiority of NNRTI-based regimens compared to PI-based regimens (Table 3 and Figure 5). However, this result does not hold for the weighted median CD4+ cell counts, for which the curves overlap. Weighted results show that only 40% and 45% of the individuals in the PI-based and NNRTI-based groups, respectively, surpass the 500 cells/μL threshold. HIV RNA viral load stratified results show no clear pattern with respect to the stratification variable but do re-enforce the finding that the observed median CD4+ cell counts are consistently higher than the weighted results (Table 3 and Figure 5). Of note is the fact that only 31% of the individuals (compared to 50%) with pretreatment viral load >100000 copies/mL (compared to < = 100000 copies/mL) surpass the >500 cells/μL threshold at year 10 (Table 3)

## Discussion

The present study contributes new data on the long-term CD4+ response to ART by contrasting observed and weighted CD4+ cell counts after start of treatment. The study population included up to ten year follow-up from two clinical cohorts from resource-rich and middle-income countries for which cohort procedures are similar and standardized. Our study thus addresses gaps that were highlighted in a recent review study including lack of information on the CD4+ response to ART after year five, for individuals who start ART at higher CD4+ cell counts (>200 cells/μL), and for populations in resource-limited settings [5]. The results show that observed (unweighted) CD4+ cell count responses were similar to those reported in studies that evaluate CD4+ cell counts among patients with HIV viral load suppression [11], [12]. The weighted results here presented, however, differed from the observed patterns showing a non-increasing CD4+ response after year four. Through the use of inverse probability of censoring weighting, we controlled for ART interruption, lost to follow-up as well as initial ART regimen indication. Thus, weighted results accurately represent the entire cohort of patients who start ART and show that observed CD4+ responses likely correspond to the optimal CD4+ response of only a subset of the population, as previously suggested [5], [7].

In addition to providing results for the entire study population, we assessed CD4+ response stratified by several socio-demographic, behavioral and clinical factors. Of all stratification variables considered in this study, the stratum with the best CD4+ response up to year ten was the subgroup of patients with pre-treatment CD4+ cell count >350 cells/μL. Only for this subgroup did three-quarters of the patients reach satisfactory immune recovery as defined by a CD4+ cell count >500 cells/μL. Similar findings have been reported in studies with shorter follow-up times from the United States [7], [11], France [12], the Netherlands [13], and resource-limited settings [14]. The present study thus corroborates and adds to these earlier results by evaluating CD4+ responses for a longer follow-up period and in a middle-income country with universal access to ART.

We found no differences in long-term CD4+ response for men and women. In fact, our results show that the apparent differences observed in the unadjusted quartiles derived from selection bias that was controlled for in the weighted results. This finding contrast with recent studies from Lao People's Democratic Republic, sub-Saharan Africa and South Africa [15]–[17]. In particular, the latter study also employed IPCW to control for lost to follow-up and found that women had a higher CD4+ response up to three years since start of ART [16]. However, it is important to note that the quartiles for the CD4+ cell counts estimates provided for both genders overlap, as do the estimates from the present study. Here, a particular gender could be said to show an improved CD4+ response at a given year (i.e. men at year 4 and women at year 5) if we chose to value the medians over the ranges. Also, the longer follow-up of the present study might explain the apparent discrepancy between the results.

The worst CD4+ response was that of IDUs or hepatitis C co-infected individuals, which were very similar. Hepatitis C co-infection and IDU are strongly correlated, 75% of the individuals who reported IDU were also hepatitis C co-infected. The use of IPCW to adjust for selection bias arising from loss to follow-up and ART discontinuation suggests that other factors are at play in determining the poor CD4+ response in this subgroup of patients. Studies have shown that the CD4+ cell count is depressed with hepatitis C co-infection and liver disease [18]-[20]. IDU is also associated with poorer ART adherence [21] which could result in a poorer CD4+ response.

We explored other socio-demographic and clinical stratification variables including age, race, AIDS defining illness (ADI), pretreatment viral load and initial ART regimens. Differently from some studies [22]–[24] but similar to other studies [25]–[27], we found that individuals with 50 years or more showed better CD4+ recovery over time. Older age has been shown to impact T cell function [28] and better adherence has been reported for older age groups [29] and this may explain the improved CD4+ response in those older than 50 years found in our study. Our study showed a poorer CD4+ response for non-whites. Studies have shown that neutropenia is more common in Blacks than Whites [30], although the proliferative response to recall antigen of peripheral blood lymphocytes may be greater in Blacks than Whites [31]. Again, adherence may play a role in the differences by race found in our study [29]. Finally, although observed results suggested that there were CD4+ cell count differences by cohort, the weighted results did not show a difference between middle-income and resource rich settings with access to ART.

Our study has strengths and limitations that are worth mentioning. A major strength of the study was the use of IPCW to correct long-term CD4+ cell count estimates for selection bias. By using this approach, we were able to study long-term CD4+ cell count response in the entire population and not for only a subset of individuals with optimal response. Other strengths include the availability of ART in both clinical cohorts since 1996 and standardized data collection of CD4+ cell counts and other measurements allowing us to directly compare long-term CD4+ cell count response among individuals followed in clinical cohorts from these two settings. Potential limitations of the analysis include the evolution of ART guidelines in both countries such that the subset of patients who initiated therapy were more likely to do so at higher CD4+ cell counts in later years [32], [33]. That said, the impact of such changes might have been minimal as we have shown that patients continue to present late for care in both settings [9]. Additionally, ART drugs and regimens have, over time, had progressively less adverse effects while fixed-dose combinations were made available primarily in the United States. Although we adjusted for whether the ART starting regimen was NNRTI- or PI-based, we were unable to adjust for the use of combination formulations that could have impacted adherence. Finally, although we used IPCW to generate unbiased estimates after adjustment for all measured confounders, as with any observational cohort, unmeasured confounding factors might have been present and this could have influenced our findings.

In conclusion, we have shown that observed CD4+ cell counts appear to increase up to 10 years from starting ART, while IPCW-weighted corrected results were more conservative, showing plateaus after the fourth year. We have also shown that the subgroup of patients with pre-treatment CD4+ cell count >350 cells/μL had the best CD4+ cell count response up to ten years. For most other subgroups evaluated, median CD4+ cell counts increased up to year 4, followed by plateaus thereafter. Moreover, the plateaus for the median CD4+ cell count were below the >500 cells/μL threshold indicating that less than 50% of the individuals had CD4+ cell counts above the lower limit of normality. The present study thus corroborates the growing body of knowledge advocating early start of ART by showing that only patients who start ART early fully recover to normal CD4+ cell count.
